# Association between Elevated De Ritis Ratio and Mortality Outcome in Adult Patients with Thoracoabdominal Trauma

**DOI:** 10.3390/healthcare10102082

**Published:** 2022-10-19

**Authors:** Wei-Ti Su, Cheng-Shyuan Rau, Sheng-En Chou, Ching-Hua Tsai, Hang-Tsung Liu, Shiun-Yuan Hsu, Ching-Hua Hsieh

**Affiliations:** 1 Department of Trauma Surgery, Kaohsiung Chang Gung Memorial Hospital, Chang Gung University College of Medicine, Kaohsiung 83301, Taiwan; 2 Department of Neurosurgery, Kaohsiung Chang Gung Memorial Hospital, Chang Gung University College of Medicine, Kaohsiung 83301, Taiwan

**Keywords:** mortality, thoracoabdominal trauma, aspartate aminotransferase (AST), alanine aminotransferase (ALT), De Ritis ratio

## Abstract

The De Ritis ratio is widely used to differentiate various causes of liver disease and serves as an independent prognostic predictor for different malignancies and non-malignant illnesses. This retrospective study aimed to identify the association between the De Ritis ratio on admission and mortality outcomes in adult thoracoabdominal trauma patients. A total of 2248 hospitalized adult trauma patients with thoracoabdominal injury, defined as an abbreviated injury scale (AIS) score ≥ 1 in the thoracic and abdominal regions, between 1 January 2009, and 31 December 2019, were included. They were categorized into three tertile groups according to the De Ritis ratio. A 1:1 propensity score-matched study group was established to attenuate the confounding effect of patient characteristics on the mortality outcome assessment. The AST levels of the tertile 1, 2, and 3 groups were 115.8 ± 174.9, 115.7 ± 262.0, and 140.5 ± 209.7 U/L, respectively. Patients in the tertile 3 group had a significantly higher level of AST than those in the tertile 1 group (*p* = 0.032). In addition, patients in the tertile 1 group had a significantly higher level of ALT than those in the tertile 2 and 3 groups (115.9 ± 158.1 U/L vs. 74.5 ± 107.0 U/L and 61.9 ± 86.0 U/L, *p* < 0.001). The increased De Ritis ratio in trauma patients with thoracoabdominal injuries was mainly attributed to elevated AST levels. The propensity score-matched patient cohorts revealed that the patients in the tertile 3 group presented a 3.89-fold higher risk of mortality than the patients in the tertile 2 group. In contrast, the patients in the tertile 1 group did not have a significantly different mortality rate than those in the tertile 2 group. This study suggests that a De Ritis ratio > 1.64 may be a useful biomarker to identify patients with a higher risk for mortality.

## 1. Introduction

Thoracoabdominal trauma is one of the main causes of mortality in trauma patients, and abdominal and thoracic trauma injuries are the second and third most common causes, respectively, of mortality in polytrauma patients [1]. For those patients with thoracoabdominal trauma, older age, higher Injury Severity Score (ISS), lower Glasgow Coma Scale (GCS) score, massive blood transfusion, initial hypotension, injuries to the liver, heart, and abdominal great vessels were identified as independent risk factors for mortality [2]. In a study of 1661 patients with thoracoabdominal trauma, the mortality rate after excluding patients with severe head trauma was 4.5% [2]. However, when patients sustained severe thoracoabdominal trauma, the 30-day mortality rate increased to 42.5% [3]. Therefore, the identification of patients at a high risk of mortality among those with thoracoabdominal trauma is important.

Aspartate aminotransferase (AST) and alanine aminotransferase (ALT) are well-known liver enzymes and blood-based circulating biomarkers [4,5] that are widely used to find out liver function in clinical settings and identify liver diseases such as viral hepatitis and alcohol addiction [4,5,6,7]. While ALT is predominantly detected only in the liver, AST is widely noticed in many organs, such as the liver, heart, kidney, brain, and skeletal muscle [8]; therefore, the contributions of these two enzymes may differ in various illnesses and can be used to distinguish among organ disorders, as ALT specifically indicates liver disease, whereas AST is associated with other organs affected in many illnesses [8]. The AST/ALT ratio, also called the De Ritis ratio [9], was first proposed in 1957 to differentiate various causes of liver disease [8,10,11]. Furthermore, the De Ritis ratio has been demonstrated to be an independent prognostic predictor for many kinds of malignancies [12,13,14,15] and non-malignant diseases, such as nonalcoholic fatty liver disease (NAFLD) [8,16], heart diseases [17,18,19], acute kidney injury [19,20,21], sepsis [22], and even in patients with COVID-19 [23,24,25,26].

Although the ratio of ALT to some other liver-related enzymes, such as gamma-glutamyl transpeptidase (GGT), alkaline phosphatase (ALP), and glutamate dehydrogenase activity (GDH) had been widely investigated, the only enzyme ratio that has proven acceptable and is still widely used is the De Ritis ratio [8]. However, the De Ritis ratio has not yet been undertaken in the trauma population, except for two studies demonstrating that the De Ritis ratio is useful for predicting survival in patients with major burn injuries [27] and following burn surgery [28]. Therefore, this retrospective study was designed to identify the association between the De Ritis ratio on admission and mortality outcomes in adult thoracoabdominal trauma patients.

## 2. Materials and Methods

### 2.1. Study Population and Data Collection

**Figure 1:**
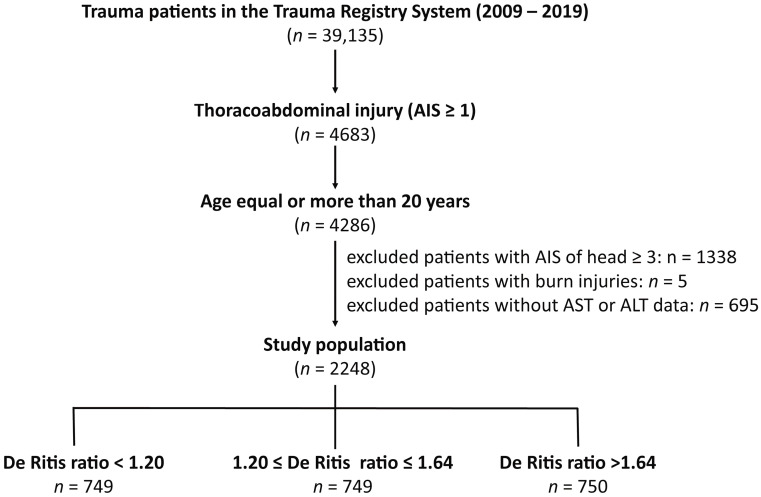
Flowchart illustrating the inclusion of hospitalized adult thoracoabdominal injury patients from the registered trauma database, with the assignment of the study patient populations into three groups according to the De Ritis ratio.

The Institutional Review Board (IRB) of Chang Gung Memorial Hospital had approved the study with the approval number 202100842B0 before the implementation of the study. Due to the retrospective design of this study, the requirement for informed consent was waived by the IRB. In this study, only hospitalized adult trauma patients with thoracoabdominal injury, defined as an abbreviated injury scale (AIS) ≥ 1 in the thoracic and abdominal regions between 1 January 2009 and 31 December 2019, were included. To attenuate the potentially lethal outcome associated with concurrent traumatic brain injury, those with head AIS ≥ 3 and patients with burns, and those lacking AST or ALT data were not included in the study. In this study, of the 39,135 trauma patients hospitalized for treatment, 4683 had thoracoabdominal injuries (Figure 1). Among these, 4286 adult patients with age ≥ 20 years were included. After excluding patients with head AIS ≥ 3 (*n* = 1338), burn injuries (*n* = 5), and incomplete AST or ALT data (*n* = 695), 2248 adult patients with thoracoabdominal injuries were included in the study population. The study population was grouped into three tertile groups, an approach commonly used in the literature [17,23,29,30,31], according to the De Ritis ratio (tertile 1 group, <1.20, *n* = 749; tertile 2 group, 1.20–1.64, *n* = 749; and tertile 3 group, > 1.64, *n* = 750). The medical information of the patients was collected from the registered trauma database of the hospital [32,33,34]. These included sex, age, preexisting comorbidities, ISS, serum AST and ALT levels (U/L) on admission, De Ritis ratio, and in-hospital mortality. The patients’ illnesses of diabetes mellitus (DM), hypertension (HTN), coronary artery disease (CAD), cerebrovascular accident (CVA), congested heart failure (CHF), and end-stage renal disease (ESRD) were considered as those preexisting comorbidities.

### 2.2. Statistical Analysis

In this study, the statistical software of Windows SPSS version 23.0 (IBM Inc., Chicago, IL, USA) was used for all statistical analyses. Two-sided Fisher’s exact test or Pearson’s χ^2^ test was used to analyze the categorical data. The normalization of the distributed continuous was evaluated by the Kolmogorov–Smirnov test. For continuous data with a normal distribution, the analysis of variance was performed with Bonferroni post hoc correction, while the Mann–Whitney *U* test was used to analyze the non-normally distributed continuous data. The continuous and noncontinuous data are expressed as mean ± standard deviation or median with interquartile range (IQR; Q1–Q3), respectively. To attenuate the confounding effect of patients’ characteristics, such as sex, age, pre-existing comorbidities, and injury severity, on the mortality outcome assessment, a 1:1 propensity score-matched study group using the Greedy method with a 0.2 caliper width, was created for the comparison of the patients who had a De Ritis ratio > 1.64 (tertile 3 group) or De Ritis ratio < 1.2 (tertile 1 group) with the patients with a De Ritis ratio of 1.2–1.64 (tertile 2 group). In this study, the primary outcome of this study was in-hospital mortality. The cut-off value of the De Ritis ratio that could predict the mortality risk of the studied population was calculated by plotting the receiver operating characteristic (ROC) curves, and the predictive performance was determined according to the area under the receiver operating characteristic curve (AUC). The maximal Youden index (defined as sensitivity + specificity − 1) was performed to determine the accuracy of the parameter in predicting mortality outcomes. In the condition of a two-tailed *p*-value < 0.05, the analyses are considered significant.

## 3. Results

### 3.1. Patient and Injury Characteristics

As shown in Table 1, there were significantly more male patients in the tertile 1 and tertile 3 groups than in the tertile 2 group. More patients with a significantly younger age were found in the tertile 1 group than in the other two groups (*p* < 0.001). Regarding the liver enzymes, there was no significant difference in the AST level between the patients in the tertile 1 group (115.8 ± 174.9 U/L), tertile 3 group (140.5 ± 209.7 U/L), and those in the tertile 2 group (115.7 ± 262.0 U/L); however, patients in the tertile 3 group had a significantly higher level of AST than those in the tertile 1 group (140.5 ± 209.7 vs. 115.8 ± 174.9 U/L, *p* = 0.032). In addition, patients in the tertile 1 group had significantly higher levels of ALT than patients in the tertile 2 and tertile 3 groups (115.9 ± 158.1 U/L vs. 74.5 ± 107.0 U/L and 61.9 ± 86.0 U/L, *p* < 0.001); however, there was no significant difference in the ALT levels between the patients in the tertile 3 group (61.9 ± 86.0 U/L) and tertile 2 group (74.5 ± 107.0 U/L). These results implied that an increased De Ritis ratio could be attributed to a higher AST level. No significant differences in preexisting comorbidities among the three groups of patients were observed. A significantly higher ISS was observed in patients in the tertile 3 group than in the tertile 2 group (median [IQR, Q3–Q3]: 13 [9,10,11,12,13,14,15,16,17,18] vs. 13 [8,9,10,11,12,13,14,15,16,17,18], respectively; *p* < 0.001). In addition, a significantly higher ISS was observed in patients in the tertile 2 group than in the tertile 1 group (median [IQR]: 13 [8,9,10,11,12,13,14,15,16,17,18] vs. 10 [8,9,10,11,12,13,14,15,16], respectively; *p* < 0.001). When the injury severity of patients was stratified by an ISS of 16–24 or an ISS ≥ 25, there were no significant differences in patient proportions among these groups; however, significantly more patients in the tertile 1 group had an ISS of 1–15, but fewer had an ISS of 16–24 than those in the tertile 2 group. The patients in the tertile 3 group had a significantly higher mortality rate than those in the tertile 2 group (3.7% vs. 1.3%, *p* = 0.004), but there was no difference in mortality between patients in the tertile 1 and tertile 2 groups (1.6% vs. 1.3%, *p* = 0.988).

### 3.2. Adjusted Outcomes of the Propensity Score-Matched Patients

After 1:1 propensity score-matched analysis, 647 and 604 well-balanced pairs of patients were selected from the tertile 3 and tertile 1 groups, respectively, versus the tertile 2 group (Table 2 and Table 3, respectively). Both propensity score-matched patient cohorts revealed no significant differences in sex, age, comorbidities, and ISS. The patients in the tertile 3 group presented with a 3.89-fold higher risk of mortality (95% confidence interval [CI] 1.44–10.50; *p* = 0.004) than those in the tertile 2 group. In contrast, the patients in the tertile 1 group did not have a significantly different mortality rate from those in the tertile 2 group (odds ratio [OR], 1.85; 95% CI 0.68–5.03, *p* = 0.222).

### 3.3. ROC Curve Analysis

**Figure 2:**
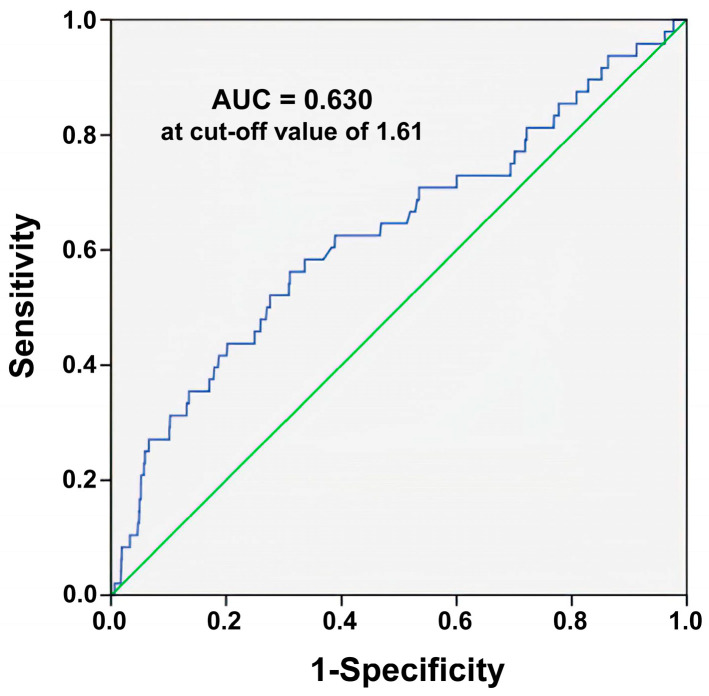
Identification of the cut-off values for predicting mortality based on the De Ritis ratio using the receiver operating characteristic curve analysis. Blue line is the plotted line of the De Ritis ratio; Green line indicates the reference line when AUC is equal to 0.5.

The ROC curve analysis (Figure 2) determined a De Ritis ratio of 1.61 is the best cut-off value for predicting mortality outcomes, with AUCs of 0.630, 68.51% accuracy, 56.3% sensitivity, and 69.0% specificity. The best cut-off value of 1.61, identified from the ROC curve, is close to the cut-off value of 1.64, which defines the patients assigned to the tertile 3 group. Although the mortality prediction relying solely on the De Ritis ratio is not good, it is better than that with an uninformative classifier yielding 0.5.

## 4. Discussion

Although the simple limit of the De Ritis ratio has been determined for clinical decisions in some illnesses (e.g., >2.0 for alcoholic hepatitis [35,36]), there are no commonly accepted reference intervals for the De Ritis ratio. In addition, the healthy limits for this ratio are yet to be ascertained. The useful cutoff value of the De Ritis ratio may depend on the studied illness. This study demonstrated that thoracoabdominal trauma patients with a De Ritis ratio > 1.64 were associated with a 3.89-fold higher risk of mortality than the propensity score-matched patient cohort in the tertile 2 group, while there was no significant difference in mortality rate between the patients in the tertile 1 and tertile 2 groups. Similar reports have been found in those articles published by Yin et al., who studied the mortality rate for adult patients with secondary hemophagocytic lymphohistiocytosis [29], by Zinellu et al., who investigated the survival probability of patients with COVID-19 during hospitalization [23], and by Lu et al., who surveyed the ICU mortality and unfavorable neurological outcome of patients with cardiac arrest [17]. Furthermore, although the patients in the tertile 1 group had significantly higher levels of ALT than those patients in the tertile 2 and tertile 3 groups, the patients in the tertile 1 group did not have a significantly different mortality rate from those in the tertile 2 group, implying that an increased AST/ALT ratio but not elevated ALT level was associated with worse patient outcomes. Therefore, for patients with thoracoabdominal trauma, a De Ritis ratio > 1.64 may be a useful biomarker to identify patients with a higher risk of sustaining mortality.

In thoracoabdominal trauma, the De Ritis ratio relies on changes in the AST and ALT levels during injury. Notably, ALT is present only in the cytoplasm of hepatocytes, whereas AST is found in both the cytoplasm and mitochondria of the hepatocytes [8]. Although the functions of both transaminases speak for important metabolic links between proteins and carbohydrates, ALT is involved in the glucose-alanine cycle to produce glucose to encounter glucose consumption, while AST plays a vital role in aerobic glycolysis [8]. For liver injury, when the death of hepatocytes is increased beyond the usual background level, the serum level of AST would indicate the cellular condition where AST is more than twice that of ALT [37]. However, only a few patients with thoracoabdominal injuries may have sustained liver injury. In a study of 1661 patients with thoracoabdominal trauma, the overall incidence of solid organ injury within the abdomen was 59.7% [2]. Considering that ALT is detected predominantly only in the liver and AST is broadly released from many organs such as the liver, heart, kidney, brain, and skeletal muscle [8], the increased level of AST may be attributed to injury to other organs or to the response of these organs against the stress associated with the trauma injury.

In this study, we used propensity score-matched patient cohorts to attenuate the effect of differences in sex, age, comorbidities, and ISS on the mortality of the patients; however, the matched patients in the tertile 3 group still presented with an around four-fold higher risk of mortality than those in the tertile 2 group, indicating that the De Ritis ratio helps to stratify the thoracoabdominal injuries in patients with a high risk for mortality. Nonetheless, this study had some limitations that should be mentioned. First, some selection bias may exist in the retrospective nature of the study. In addition, a selection bias may exist in the outcome measurement since this study only assessed in-hospital mortality but not long-term mortality, and some patients were excluded without data for AST and ALT levels. Furthermore, the presence of undetected liver disease may exist in the study population cohort, leading to a change in the De Ritis ratio, regardless of thoracoabdominal trauma. Moreover, some interventions such as damage control, resuscitation, and operation may result in different outcomes for the patients and were not controlled for further analysis. However, we can only assume that the outcomes of these interventions were uniform across the studied patient population. Finally, the results of this study were limited to a single urban trauma center and may not be generalizable to other areas.

## 5. Conclusions

This study demonstrated that the De Ritis ratio may be a useful tool to recognize the high mortality risk in adult patients with thoracoabdominal trauma. The investigation of the mechanism behind the AST and ALT changes upon trauma injury would help a more precise application of the De Ritis ratio in the clinical setting. In addition, it would be interesting to study whether this ratio could be used in the stratification of major complications other than mortality and used for patients with trauma injuries other than thoracoabdominal trauma.

## Figures and Tables

**Table 1:** Patient and injury characteristics of the study population according to the De Ritis ratio.

	De Ritis Ratio			
Variables	<1.20 (Tertile 1) *n* = 749	1.20–1.64 (Tertile 2) *n* = 749	>1.64 (Tertile 3) *n* = 750	*p*
Gender				0.001
Male, n (%)	558 (69.8) *	474 (61.2)	456 (67.7) *	
Female, n (%)	242 (30.2) *	300 (38.8)	218 (32.3) *	
Age (years)	47.0 ± 16.4 *	50.4 ± 17.6	49.9 ± 18.1	<0.001
AST (U/L)	115.8 ± 174.9	115.7 ± 262.0	140.5 ± 209.7	0.049
ALT (U/L)	115.9 ± 158.1 *	74.5 ± 107.0	61.9 ± 86.0	<0.001
Comorbidities				
DM, n (%)	104 (13.0)	88 (11.4)	79 (11.7)	0.580
HTN, n (%)	181 (22.6)	160 (20.7)	160 (23.7)	0.361
CAD, n (%)	16 (2.0)	20 (2.6)	13 (1.9)	0.633
CVA, n (%)	7 (0.9)	12 (1.6)	16 (2.4)	0.068
CHF, n (%)	2 (0.2)	4 (0.5)	5 (0.7)	0.399
ESRD, n (%)	10 (1.2)	4 (0.5)	6 (0.9)	0.301
ISS, median (IQR)	10 (8–16) *	13 (8–18)	13 (9–18) *	<0.001
1–15, n (%)	581 (72.6) *	508 (65.6)	413 (61.3)	<0.001
16–24, n (%)	161 (20.1) *	196 (25.3)	189 (28.0)	0.001
≥25, n (%)	58 (7.2)	70 (9.0)	72 (10.7)	0.069
Mortality, n (%)	13 (1.6)	10 (1.3)	25 (3.7) *	0.003

ALT, alanine aminotransferase; AST, aspartate aminotransferase; CAD, coronary artery disease; CHF, congestive heart failure; CI, confidence interval; CVA, cerebral vascular accident; DM, diabetes mellitus; ESRD, end-stage renal disease; GCS, Glasgow Coma Scale; HTN, hypertension; IQR, interquartile range; ISS, injury severity score; OR, odds ratio. * indicated *p* < 0.05 when compared with patients with a De Ritis ratio between 1.20–1.64 (Tertile 2 group).

**Table 2:** Propensity score matched-cohort of patients in the tertile 3 vs. tertile 2 groups.

Propensity Score Matched-Cohort								
	De Ritis Ratio				OR (95% CI)		*p*	Standardized Difference
	>1.64 (Tertile 3) *n* = 604		1.20–1.64 (Tertile 2) *n* = 604					
Male, *n* (%)	403	(66.7)	403	(66.7)	1.00	(0.79–1.27)	1.000	0.00%
Age (years)	48.9	±17.9	48.7	±18.0	-		0.839	1.17%
DM, *n* (%)	59	(9.8)	59	(9.8)	1.00	(0.68–1.46)	1.000	0.00%
HTN, *n* (%)	126	(20.9)	126	(20.9)	1.00	(0.76–1.32)	1.000	0.00%
CAD, *n* (%)	8	(1.3)	8	(1.3)	1.00	(0.37–2.68)	1.000	0.00%
CVA, *n* (%)	6	(1.0)	6	(1.0)	1.00	(0.32–3.12)	1.000	0.00%
CHF, *n* (%)	1	(0.2)	1	(0.2)	1.00	(0.06–16.02)	1.000	0.00%
ESRD, *n* (%)	0	(0.0)	0	(0.0)	-		-	-
GCS	15	(15–15)	15	(15–15)	-		0.479	−4.07%
ISS	13	(9–18)	13	(9–18)	-		0.966	−0.24%
Mortality	19	(3.1)	5	(0.8)	3.89	(1.44–10.50)	0.004	-

CAD, coronary artery disease; CHF, congestive heart failure; CI, confidence interval; CVA, cerebral vascular accident; DM, diabetes mellitus; ESRD, end-stage renal disease; HTN, hypertension; IQR, interquartile range; ISS, injury severity score; OR, odds ratio.

**Table 3:** Propensity score matched-cohort of patients in the tertile 1 vs. tertile 2 groups.

Propensity Score Matched-Cohort								
	De Ritis Ratio				OR (95% CI)		*p*	Standardized Difference
	<1.20 (Tertile 1) *n* = 647		1.20–1.64 (Tertile 2) *n* = 647					
Male, *n* (%)	426	(65.8)	426	(65.8)	1.00	(0.80–1.26)	1.000	0.00%
Age (years)	48.0	±16.2	48.6	±16.7	-		0.539	−3.42%
DM, *n* (%)	71	(11.0)	71	(11.0)	1.00	(0.71–1.42)	1.000	0.00%
HTN, *n* (%)	136	(21.0)	136	(21.0)	1.00	(0.77–1.31)	1.000	0.00%
CAD, *n* (%)	9	(1.4)	9	(1.4)	1.00	(0.39–2.54)	1.000	0.00%
CVA, *n* (%)	4	(0.6)	4	(0.6)	1.00	(0.25–4.02)	1.000	0.00%
CHF, *n* (%)	0	(0.0)	0	(0.0)	-	-	-	
ESRD, *n* (%)	2	(0.3)	2	(0.3)	1.00	(0.14–7.12)	1.000	0.00%
GCS	15	(15–15)	15	(15–15)	-		0.655	−2.48%
ISS	11	(8–17)	12	(8–17)	-		0.703	−2.12%
Mortality	11	(1.7)	6	(0.9)	1.85	(0.68–5.03)	0.222	-

CAD, coronary artery disease; CHF, congestive heart failure; CI, confidence interval; CVA, cerebral vascular accident; DM, diabetes mellitus; ESRD, end-stage renal disease; GCS, Glasgow Coma Scale; HTN, hypertension; ISS, injury severity score; OR, odds ratio.

## Data Availability

Not applicable.
